# Accuracy Tests of a Dual-Class Hybrid FBG/PZT Photonic Current Transducer Featuring a Novel Passive Autoranging Circuit

**DOI:** 10.3390/s26020663

**Published:** 2026-01-19

**Authors:** Burhan Mir, Grzegorz Fusiek, Pawel Niewczas

**Affiliations:** Department of Electronic and Electrical Engineering, University of Strathclyde, Glasgow G1 1XQ, UK; g.fusiek@strath.ac.uk (G.F.); p.niewczas@strath.ac.uk (P.N.)

**Keywords:** autoranging, zero crossing, fiber Bragg grating, piezoelectric transducer, photonic current transducer

## Abstract

This paper reports, for the first time, the characterization and measurement accuracy evaluation of a photonic current transducer (PCT) featuring a hybrid fiber Bragg grating/piezoelectric transducer (FBG/PZT) and an integrated passive autoranging (AR) circuit. The enhanced sensor is designed to meet both metering-class (0,2 S) and protection-class (5P15) requirements simultaneously—capabilities not yet demonstrated by any other device in the industry that also supports remote interrogation and multiplexing of multiple sensors. The autoranging technique employs MOSFET switches to dynamically adjust the burden resistance, preventing FBG/PZT voltage saturation during fault or thermal-current events while maintaining adequate sensitivity at lower currents. Experimental results show that integrating the PCT with the passive AR circuit significantly extends the device’s dynamic range, reduces current-measurement errors, and demonstrates potential compliance with both 0,2 S metering- and 5P15 protection-class requirements. The results also confirm that the sensor operates correctly across this extended range.

## 1. Introduction

Achieving the required accuracy in voltage and current measurements in accordance with IEEE and IEC standards is essential for effective metering and protection in power systems [1,2].

Conventional iron-core voltage transformers (VTs) and current transformers (CTs) are widely used but present notable disadvantages, including considerable weight, high installation costs, and a large substation footprint. They also lack galvanic isolation, which can pose safety risks such as electrocution, fire, and explosion [3,4,5,6].

To address these limitations, the industry is increasingly adopting non-conventional instrument transformers (NCITs), such as fiber-optic current sensors (FOCS) that employ optical-fiber communication links and digital protocols compatible with the IEC 61,850 standard, enabling the deployment of more compact, digital substations. These devices can achieve high accuracy (±0.1%) for both AC and DC measurements. Compared with traditional CTs and VTs, NCITs offer several advantages, including reduced weight, enhanced safety, lower insulation requirements, fewer environmental hazards, reduced primary power losses, and a broader bandwidth with greater dynamic range [7,8,9,10].

However, despite their benefits, optical NCITs face challenges related to multiplexing and remote interrogation, which limit their suitability for passive, long-distance deployment in wide-area network monitoring. To overcome the limitations of traditional optical NCITs and enable access to multiple remote, distributed, passive voltage and current measurements over long distances, the authors introduced innovative photonic transducers based on fiber Bragg grating (FBG) sensors and piezoelectric (PZT) components [11].

The authors previously designed an optical current sensor for protection purposes and assessed its performance against industry standards. By integrating FBGs and PZT transducers to measure voltage across conventional CT burdens or Rogowski coils, they developed an FBG/PZT-based photonic current transducer (PCT). Their studies demonstrated that the prototype PCT satisfied the 5P accuracy class. Nevertheless, the proposed FBG/PZT PCT has a limited measurement range and cannot match the dynamic range or accuracy of FOCS-based NCITs [12].

A common solution for extending dynamic range involves duplicating the measurement chain, for example, by using separate metering- and protection-class PCTs. However, this approach does not support the combined 0,2/0,2 S and 5P classes and requires two sensors, increasing size, weight, and cost. Furthermore, using two FBG/PZT sensors halves the available optical bandwidth, reducing the number of sensors that a single interrogation unit can address. To mitigate these issues, the authors introduced a novel autoranging concept for the PCT [13]. This approach employs multiple serially connected burdens and power MOSFET switches driven by a passive comparator circuit integrated within the PCT. When the voltage across a burden reaches a threshold, the circuit bypasses the current, dynamically reducing sensitivity and extending the PCT’s measurement range. This method provides advantages such as reduced size and weight, preservation of optical bandwidth, and inherent burden protection, as excess current is diverted during faults or tests, enabling compliance with industry thermal-current protection requirements. Experimental results demonstrated that the autoranging concept fills a technological gap in hybrid optical sensors by passively extending their measurement range at the remote end. Previous work presented a proof-of-concept two-stage autoranging circuit, detailing its design and providing an extensive test report. That study showed that the circuit reliably responds to emulated secondary CT current levels by bypassing two burden resistors at prescribed thresholds, offering a passive, local means of adjusting sensitivity in the PCT [14].

The autoranging circuit was tested with an FBG/PZT sensor and an FBG interrogator under various operating conditions using 1 Ω and 16 Ω burdens. The sensor was evaluated in normal, fault, and thermal-current modes. It successfully detected threshold crossings at 130% of nominal current and at 22 times the rated current within 4 ms, and it remained stable under 100 A thermal currents for 1 s, as required by some utilities. The switching algorithm enabled reliable reconstruction of burden current from optical signals and supported the development of a dual-class optical current sensor with a wide dynamic operating range. Issues related to range-change detection near the zero-crossing of the current waveform were resolved through an improved algorithm that enhanced reconstruction accuracy at the interrogator [15].

Building on this previous research [14,15,16], this paper provides, for the first time, a quantitative evaluation of the current-measurement accuracy achievable by a hybrid PCT combined with an autoranging circuit. The main goal of this contribution is to demonstrate that the proposed solution can deliver increased dynamic range and a dual-class passive sensor device, targeting potential compliance with the combined 0,2 S metering and 5P15 protection classes—capabilities that were previously not achievable with a single PCT device. 

## 2. IEC Accuracy Requirements

According to IEC standards, the metering and protection classes used by power system operators are 0,2, 0,2 S, and 5P (e.g., 5P15). The corresponding accuracy specifications are listed in Table 1 below [1,2].

The current PCT can achieve the 5P protection accuracy class with a nominal burden voltage of 1 V (rms). At present, no technology enables a PCT to meet the combined 0,2/0,2 S metering and 5P protection classes within a single device. The novel autoranging concept for PCTs, explored in the authors’ previous work [14,15,16], provides a means not only to extend the measurement range without requiring additional voltage sensors or CTs, but also to integrate both metering and protection functionalities into a single device. Since the improved sensor configuration is intended to comply with both protection and metering classes, its current-measurement errors must fall within the IEC limits specified in Table 1.

## 3. Experimental Setup and Methodology

In previous research [14,15,16], the authors presented a passive autoranging method that employed a PCT connected to multiple serially linked burden resistors, with the selection controlled by static MOSFET switches according to the measured current magnitude. A bespoke switching algorithm enabled reconstruction of the measured current from the optical signals using dedicated scaling factors. In this work, we replicate that scheme to compare the PCT’s current-measurement accuracy with and without the autoranging (AR) circuit. The details of this comparison are discussed in the following sections.

**Figure 1:**
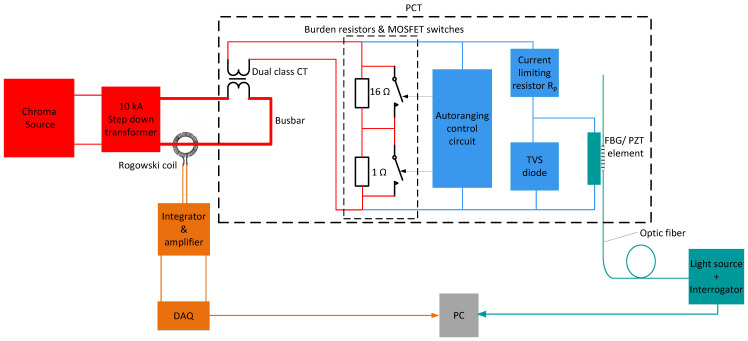
Block diagram of the experimental setup for PCT calibration and testing.

A schematic diagram of the replicated experimental setup is shown in Figure 1. 

The PCT in Figure 1 is highlighted by a large dashed rectangular box. It comprises the following elements:

1. **Dual-class CT** (Instrument Transformers Ltd.), providing combined 0,2 S metering-class and 5P protection-class accuracy. The CT converts the primary busbar current (250 A nominal) to a nominal secondary current of 1 A.
2. **CT burdens with associated MOSFET switches.** The current through the burden resistors is controlled by MOSFET switches driven by the autoranging control circuit [13,14,15,16].
3. **Autoranging (AR) circuit.** The AR control circuit uses a passive, high-speed, low-power comparator and is powered directly from the CT secondary terminals [14,15,16].
4. **FBG/PZT transducer.** A detailed description of the operating principle of the FBG/PZT transducer is provided in [11,12]. In brief, the PZT responds to the voltage across the burden by changing its longitudinal dimension. The resulting strain change is measured by the FBG physically attached to the PZT transducer. The corresponding shift in the FBG center wavelength is then calibrated to obtain current readings.
5. **Protection components.** A current-limiting resistor and a transient-voltage-suppression (TVS) diode connected across the FBG/PZT transducer are used to prevent overcurrent, depolarization, and damage during overvoltage events [12].

A Chroma 61,512 precision AC voltage source powered a 10 kA step-down transformer whose secondary winding was connected to a busbar. A Rogowski coil with a dedicated integrator/amplifier (Rocoil SQ-1311) was used as the reference current-measurement device with 0.1% accuracy. 

The FBG/PZT transducer was interrogated using an optical source and an FBG interrogator (I-MON 256 USB, Ibsen Photonics) operating at 4 kHz and connected to a personal computer (PC). The voltage signal from the Rogowski coil integrator output was acquired using an NI USB-6003 data acquisition (DAQ) card operating at 4 ksps and also connected to the PC.

The PCT sensor was calibrated and tested under laboratory conditions at a stable room temperature of 20 ± 1 °C. Current-measurement accuracy was validated by comparing the amplitudes of the optical and electrical signals. Because the I-MON unit and DAQ card do not permit precise synchronization of the optical and electrical channels, phase-shift measurements could not be performed. As a result, only amplitude errors were evaluated in accordance with IEC requirements, as explained in the next section.

To demonstrate the benefits of the autoranging technique, current-measurement errors were analyzed for two cases:

**Case 1:** The FBG/PZT was used to measure the voltage directly across a single 1 Ω burden resistor without the AR circuit. The transducer voltage range (±30 V) allowed measurement of primary currents from 0 A up to 250 A (nominal) and, in principle, up to 5000 A (20 times nominal). At 5000 A, the burden voltage would reach a peak amplitude of approximately 28 V. Due to the limitation of the step-down transformer, the maximum test current was just below 4000 A. This enabled testing up to the 5P15 protection class using currents up to 3750 A, for which the burden voltage reached a peak amplitude of approximately 21 V.

**Case 2:** The AR circuit was connected across the series combination of the 16 Ω and 1 Ω burdens and automatically scaled the FBG/PZT voltage according to the primary-current magnitude. In this configuration, almost the full FBG/PZT voltage range was used for currents up to 1.2 times nominal, reaching a peak amplitude of approximately 29 V. For fault currents, the AR circuit bypassed the 16 Ω burden resistor, which prevented excessive voltage across the FBG/PZT transducer. At 3750 A, the transducer voltage was the same as in Case 1, with a peak amplitude of 21 V.

**Figure 2:**
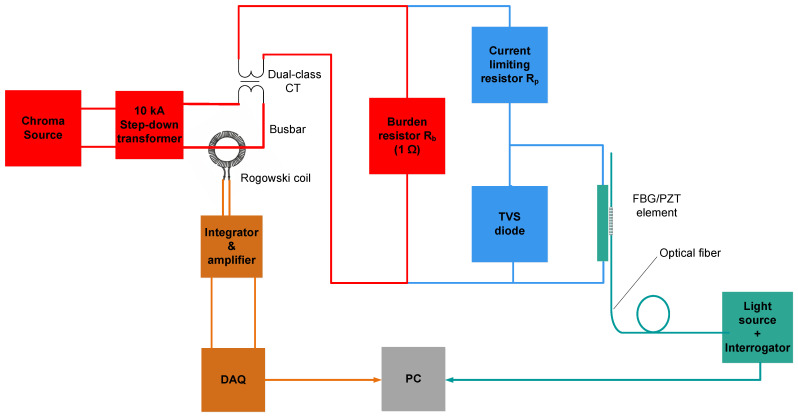
Block diagram of the experimental setup: Case 1 (sensor without autoranging circuit).

**Figure 3:**
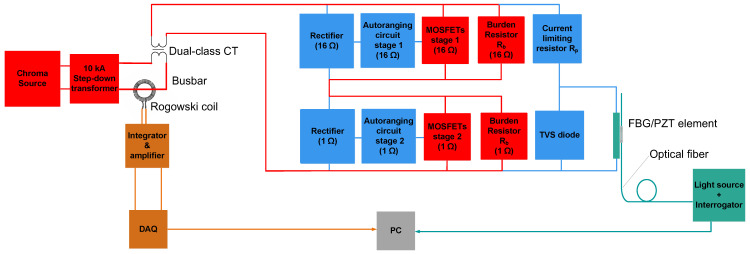
Block diagram of the experimental setup Case 2 (sensor with integrated autoranging circuit).

Figure 2 and Figure 3 show the modifications of the experimental setup from Figure 1 corresponding to Case 1 and Case 2, respectively. Note that in Figure 1, Figure 2 and Figure 3, high-current paths are shown in red, low-current paths in blue, the optical interrogation section in green, and the Rogowski coil electrical-signal interrogation section in orange.

## 4. Sensor Characterization and Accuracy Tests 

### 4.1. Sensor Characterization and Accuracy Tests Without Autoranging Circuit (1 Ω Burden, Case 1) 

To calibrate the PCT without the autoranging circuit, as shown in Figure 2, a series of AC current injections at 50 Hz was applied through the Chroma/step-down transformer source, covering 1% to 120% of the nominal current, and further extended up to 15 times the nominal current (3750 A). The testing included steps at 1%, 5%, 20%, 100%, 120%, and 15 times the nominal current, in accordance with IEC specifications.

The sampling rate for both electrical and optical signals was 4 kHz, providing 80 samples per cycle. At each current step, measurements were recorded for 1 s, covering 50 cycles (a total of 4000 samples). RMS values were calculated from data collected over 20 cycles at each current level, specifically from the 10th to the 30th cycle, to avoid potential transients at the start and end of the datasets. 

**Figure 4:**
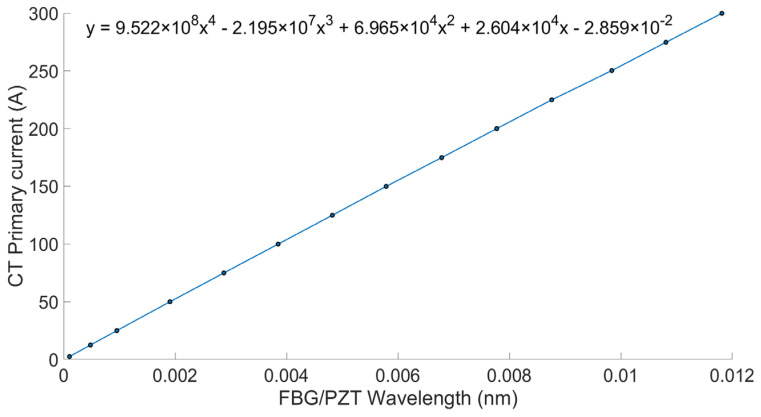
PCT calibration curve for measurements without AR circuit (Case 1).

A calibration curve for the sensor, shown in Figure 4, was created by fitting a fourth-order polynomial to the RMS data obtained during initial characterization. This dataset included reference current values and the corresponding FBG wavelength shifts over the range of 1% to 120% of nominal current. The resulting polynomial equation was integrated into the analysis software (MATLAB R2021b), enabling the conversion of FBG peak wavelength shifts into reconstructed current values.

To evaluate compliance with protection and metering accuracy standards, 50 Hz current waveforms at amplitudes of 1%, 5%, 20%, 100%, 120%, and 15 times the nominal current were used. The current amplitude error in steady-state conditions is defined as:

(1) $$\varepsilon_{i}(\%)=\frac{I_{p}-I_{rec}}{I_{p}}\times 100$$

where $I_{p}$ is the rms value of the primary or reference current and $I_{rec}$ is the rms value of the reconstructed current.

### 4.2. Sensor Characterization and Accuracy Tests with Integrated Autoranging Circuit and Combined 1 Ω and 16 Ω Burden (Case 2) 

To calibrate the PCT with the integrated autoranging circuit, as shown in Figure 3, 50 Hz AC current was injected through the Chroma/step-down transformer source over the range of 1% to 120% of the nominal current, and further extended up to 15 times the nominal current (3750 A). The testing included steps at 1%, 5%, 20%, 100%, 120%, and 15 times the nominal current, as specified by IEC. Repeated measurements of the optical signals from the FBG/PZT transducer and the electrical signals from the Rogowski coil were recorded on the PC for calibration and error analysis.

The sampling rate for both optical and electrical signals was 4 kHz, resulting in 80 samples per cycle. As before, at each injected current value, the optical and electrical readings were recorded. RMS values were calculated from 20 cycles selected between the 10th and 30th cycles to avoid potential transients at the start or end of the dataset.

**Figure 5:**
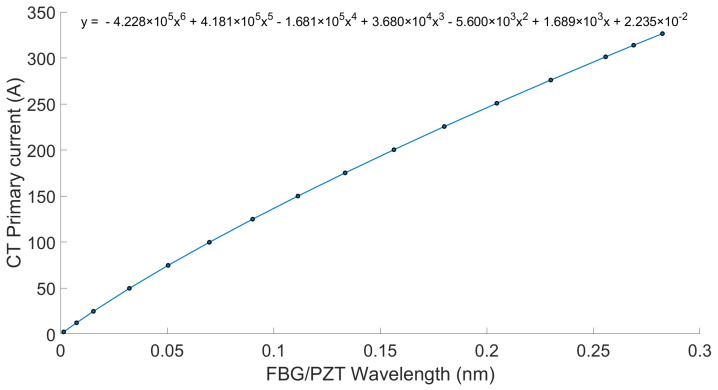
PCT calibration curve for 0,2 S metering class (Case 2 integrated AR circuit with the combined 17 Ω burden).

The calibration curve for the sensor, shown in Figure 5, was generated by fitting a sixth-order polynomial to the RMS data collected during initial characterization. A sixth-order polynomial provided a better fit than the fourth-order polynomial used in Case 1. In Case 2, the full dynamic range of the FBG/PZT transducer was utilized, resulting in a calibration curve with greater nonlinearity due to increased hysteresis in the PZT at higher voltage amplitudes. This effect is described in detail in the authors’ earlier hysteresis compensation studies [17,18]. The calibration dataset covered the reference current and wavelength shifts from 1% to 120% of nominal current. The derived polynomial equation was then implemented in the analysis software (MATLAB R2021b) to convert FBG peak wavelength shifts into reconstructed current values.

**Figure 6:**
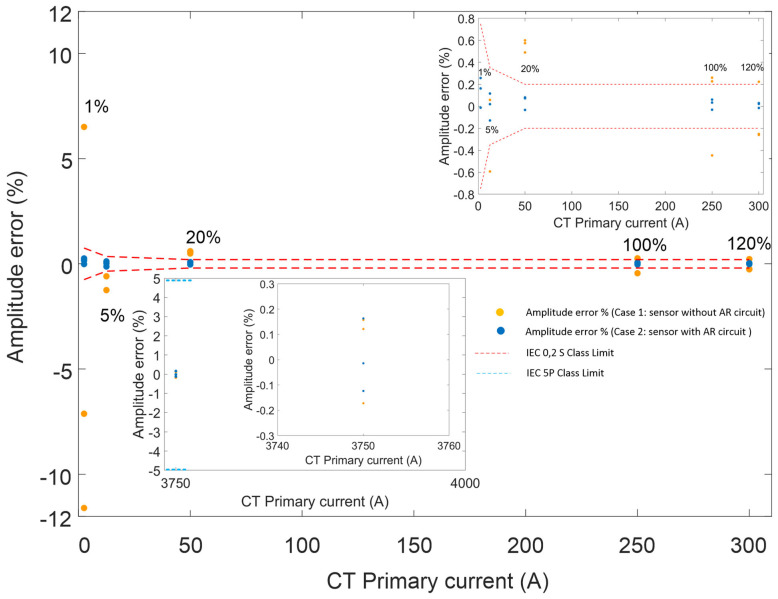
Amplitude errors for the 0,2 S metering class and the 5P15 protection class for both Case 1 (orange data points) and Case 2 (blue data points).

To assess compliance with IEC metering and protection accuracy classes, 50 Hz current waveforms at amplitudes of 1%, 5%, 20%, 100%, 120%, and 15 times the nominal current were injected. Each measurement was repeated four times. The amplitude error in steady-state conditions was calculated using (1). The results for three consecutive runs are presented in Figure 6.

Figure 6 includes two insets to aid interpretation. The main plot shows amplitude error (%) for both Case 1 and Case 2 at 1%, 5%, 20%, 100%, and 120% of nominal current, with CT primary current on the horizontal axis (0–300 A) and amplitude error on the vertical axis (−12% to 12%). The first inset, at the top right, presents the same data but with a reduced vertical scale (−0.8% to 0.8%) to highlight small deviations. The second inset, at the bottom left, focuses on the amplitude error at 15 times nominal current (3750 A) and demonstrates that both Case 1 and Case 2 comply with the IEC 5P15 protection-class limit.

Clearly, for Case 1 (orange data points in Figure 6), the PCT meets only the 5P15 protection-class requirements. In contrast, in Case 2 (blue data points), the use of the autoranging technique enables the same device to satisfy both the 0,2 S metering-class and 5P15 protection-class limits.

## 5. Discussion

Table 1 shows that for the IEC 0,2 S metering class, the current amplitude error at the rated current must remain within ±0.75% at 1% of the rated current (2.5 A). At 5% of the rated current (12.5 A), the limit is ±0.35%, while at 20%, 100%, and 120% of the rated current (50 A, 250 A, and 300 A), the error must not exceed ±0.2%. For the IEC 5P protection class, the current amplitude error at fifteen times the rated current (3750 A) must remain within ±5%.

As demonstrated in Section 3, integrating the PCT module with the autoranging circuit significantly improved the sensor’s accuracy. The data clearly show that without the autoranging circuit, the sensor does not meet the 0,2 S metering-class requirements, although it still satisfies the 5P protection-class limit. When combined with the passive autoranging circuit, the sensor successfully achieves both the 0,2 S metering class and the 5P15 protection class within a single device, representing a performance level that has not previously been demonstrated in hybrid FBG/PZT sensors. Figure 6 illustrates that with the autoranging circuit in place, the sensor’s amplitude errors remain within the IEC limits, whereas without the circuit, the errors exceed the specified thresholds. These findings clearly confirm that incorporating the autoranging circuit enhances both the performance and accuracy of the sensor. The measured amplitude errors remain comfortably within the IEC limits for both the 0,2 S metering class and the 5P15 protection class.

While the sensor remained stable during testing, further work is required to evaluate its long-term performance. Additional improvements in accuracy could be achieved by using more precise, synchronized instrumentation for reference-current measurement and sensor characterization, together with an interrogator system offering lower noise and higher wavelength resolution. A comparison of the sensor’s capabilities in both cases is presented in Table 2.

## 6. Conclusions and Future Work 

This paper has detailed the characterization and testing of an upgraded hybrid FBG/PZT PCT integrated with an autoranging circuit. Laboratory tests conducted at room temperature evaluated its performance in line with IEC standards. The current-measurement amplitude errors remained within the required limits, confirming that the improved PCT module has the potential to meet the criteria for 0,2 S metering and 5P15 protection classes within a single device.

Future work will focus on improvements to the experimental setup to enable phase-shift measurements between the electrical and optical signals and facilitate comprehensive phase-error analysis. Further studies will also investigate compensation of environmental and hysteresis effects, long-term performance, and the miniaturization and ruggedization of the PCT, as well as its integration with digital substation systems to ensure reliability, interoperability, and compatibility in modern power networks.

## Figures and Tables

**Table 1:** IEC metering- and protection-class specifications.

Accuracy Class	±Percentage Current (Ratio) Error at Percentage of Rated Current Shown Below					±Phase Error at Percentage of Rated Current Shown Below									
						Minutes					Centiradians				
	1	5	20	100	120	1	5	20	100	120	1	5	20	100	120
**0,1**	-	0.4	0.2	0.1	0.1	-	15	8	5	6	-	0.45	0.24	0.15	0.15
**0,2**	-	0.75	0.35	0.2	0.2	-	30	15	10	10	-	0.9	0.45	0.3	0.3
**0,5**	-	1.5	0.75	0.5	0.5	-	90	45	30	30	-	2.7	1.35	0.9	0.9
**1,0**	-	3.0	1.5	1.0	1.0	-	180	90	60	60	-	5.4	2.7	1.8	1.8
**IEC special application metering-class specifications**															
**0,2 S**	0.75	0.35	0.2	0.2	0.2	30	15	10	10	10	0.9	0.45	0.3	0.3	0.3
**0,5 S**	1.5	0.75	0.5	0.5	0.5	90	45	30	30	30	2.7	1.35	0.9	0.9	0.9
**IEC protection-class specifications**															
**Accuracy** **Class**	**Current Error at Rated Primary Current %**	**Phase Error at Rated Primary Current**						**Composite Error at Rated Accuracy Limit** **Primary Current** **%**				**At Accuracy Limit Condition** **Maximum Peak Instantaneous Error** **%**			
		**Minutes**			**Centiradians**										
**5TPE**	±1	±60			±1.8			5				10			
**5P**	±1	±60			±1.8			5				-			
**10P**	3	-			-			10				-			

**Table 2:** Comparison of sensor capabilities in both Case 1 and Case 2 configurations.

Sensor Configuration	Burden Resistor	Autoranging Enhancement	0,2 S Metering-Class Accuracy Achievement	5P Protection-Class Accuracy Achievement	Combined 0,2 S Metering and 5P Protection
Sensor configuration Case 1	1 Ω	✗	✗	✓	✗
Sensor configuration Case 2	17 Ω	✓	✓	✓	✓

## Data Availability

All data underpinning this publication are openly available from the University of Strathclyde KnowledgeBase at https://doi.org/10.15129/e9a4ac0e-8e25-4ab6-8b0a-5de63e5e0bdc.
